# Bilateral endogenous endophthalmitis from asymptomatic *Aspergillus niger* fungal infective endocarditis in the immunosuppressed patient: A case report

**DOI:** 10.1097/MD.0000000000046651

**Published:** 2025-12-19

**Authors:** Ji Min Kim, Seowoo Park, Jin-Ho Joo

**Affiliations:** a Department of Pathology, Ewha Womans University Mokdong Hospital, Ewha Womans University College of Medicine, Seoul, Republic of Korea; b Department of Ophthalmology, Uijeongbu St. Mary’s Hospital, College of Medicine, The Catholic University of Korea, Seoul, Republic of Korea; c Department of Ophthalmology, Chung-Ang University College of Medicine, Seoul, Republic of Korea; d Department of Ophthalmology, Chung-Ang University Gwangmyeong Hospital, Gwangmeyong-si, Republic of Korea.

**Keywords:** *Aspergillus niger*, case report, endogenous endophthalmitis, fungal endocarditis, immunosuppression

## Abstract

**Rationale:**

Endogenous fungal endophthalmitis (EFE) is a rare but sight- and life-threatening condition that often arises in immunocompromised patients. While *Aspergillus fumigatus* is the predominant pathogen, *Aspergillus niger*-associated endocarditis leading to bilateral EFE has not been previously reported.

**Patient concerns:**

A 51-year-old woman with a history of two renal transplants over the past 30 years, along with diabetes mellitus, hypertension, and ongoing immunosuppressive therapy, presented with rapidly progressive bilateral visual impairment, beginning with acute vision loss and ocular pain in the right eye, followed by involvement of the left eye within two days. She had no systemic symptoms such as fever, chest pain, or dyspnea.

**Diagnosis:**

Initial aqueous humor analysis, including Gram staining, fungal culture, and viral PCR, yielded negative results. As the disease progressed to bilateral involvement within 2 days, a diagnostic vitrectomy was performed. Vitreous sampling demonstrated fungal hyphae, and culture confirmed *Aspergillus niger*. Transesophageal echocardiography further revealed mitral valve vegetation, establishing the diagnosis of fungal infective endocarditis with bilateral endogenous endophthalmitis.

**Interventions:**

The patient received empiric intravitreal antibiotics and antivirals initially, followed by diagnostic pars plana vitrectomy and systemic antifungal therapy after identification of *Aspergillus*. Mitral valve replacement surgery was subsequently performed.

**Outcomes:**

Despite surgical intervention and systemic antifungal treatment, the patient developed disseminated fungal sepsis and died 2 weeks after valve replacement.

**Lessons:**

This case highlights the diagnostic challenges of EFE and emphasizes the importance of considering fungal endocarditis as a source of infection in immunosuppressed patients presenting with severe vitritis, even in the absence of systemic symptoms. Early recognition, prompt vitrectomy with intravitreal antifungal therapy, and thorough systemic evaluation are essential to improve visual and systemic outcomes.

## 1. Introduction

Endophthalmitis is an infection of the vitreous and/or aqueous humor by bacteria or fungi, which can be fatal to the eye and cause irreversible blindness. Endogenous endophthalmitis (EE) is caused by the hematogenous seeding of pathogens during bacteremia or fungemia and occurs in 5% to 15% of all cases of endophthalmitis. EE is known to be more common in people with advanced age, indwelling catheters, intravenous drug use, or relative immunosuppression such as age, malignancy, diabetes mellitus, or use of immunosuppressive agents.^[1]^

If the underlying disease causing the infection is unknown, it is important to identify the causative agent of EE. EE is diagnosed by culture of aqueous humor and/or vitreous fluid and blood culture. However, cultures are negative in 20% to 30% of cases, so if the diagnosis is not made or treatment is delayed, vision loss can be irreversible, and in severe cases, the patient may die. EE is a medical emergency and requires immediate diagnosis and treatment to preserve vision.^[2]^ However, because there are no randomized clinical trials for EE, there is no universally accepted protocol for diagnosis and treatment.

The following case is a report of an immunosuppressed patient with asymptomatic fungal endocarditis who presented with blurred vision and died due to a delayed diagnosis. Written informed consent for publication could not be obtained because the patient was deceased. Permission from the patient’s relatives was not possible; therefore, the Institutional Review Board of Uijeongbu St. Mary’s Hospital (UC22ZISI0139) approved the publication of this case report and took responsibility for anonymization of the patient data.

## 2. Case report

A 51-year-old woman with a history of two renal transplants over the past 30 years presents to the ophthalmology department complaining of decreased vision and pain in her right eye that started 1 day earlier. She had a past medical history of diabetes mellitus, hypertension, and cytomegalovirus (CMV) pneumonia treated 6 months ago. The patient was on immunosuppressive therapy with 3 mg of tacrolimus and 6 mg of deflazacort. There were no symptoms such as fever, chest pain, or dyspnea.

**Figure 1.**
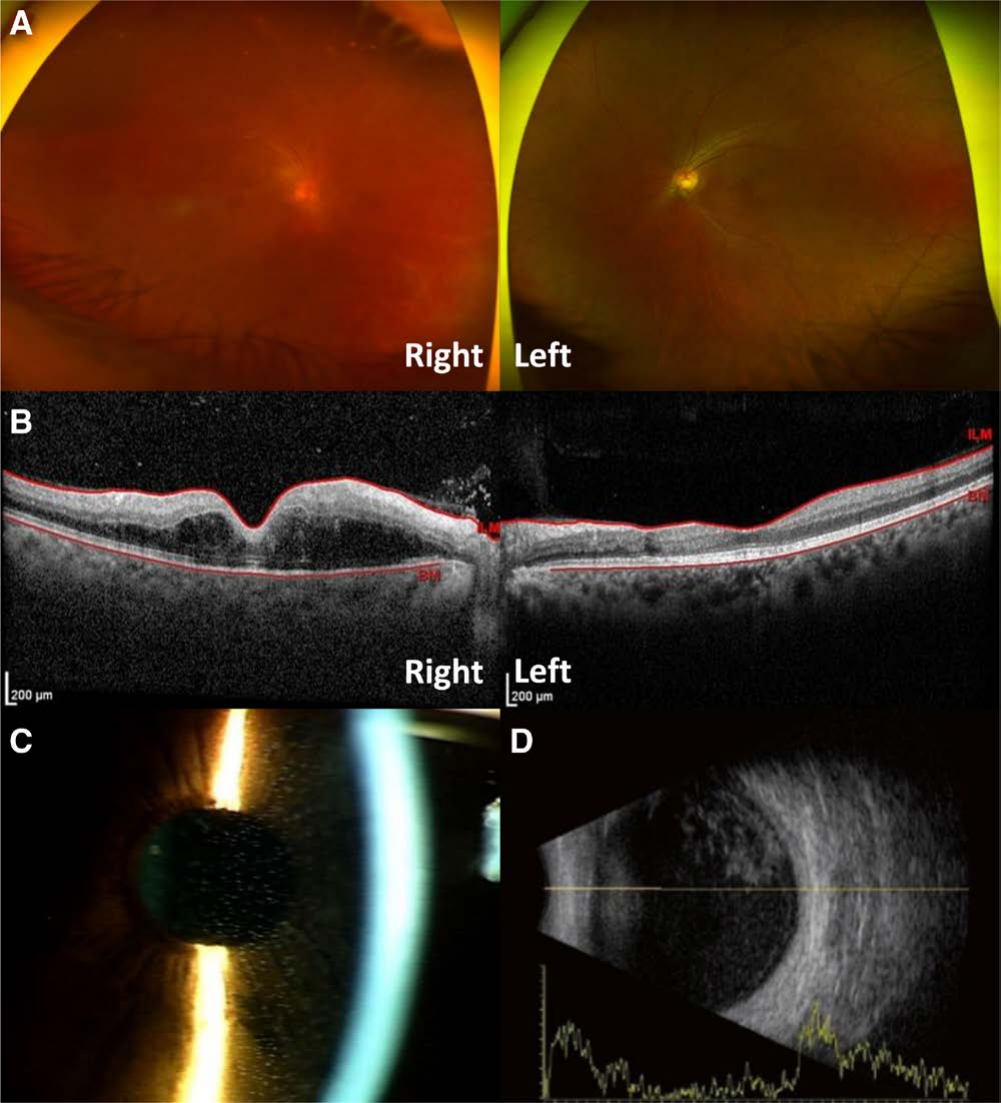
Fundus examination, optical coherence tomography, and B-scan ultrasonography the first day of a patient`s visit for decreased vision in the right eye. (A) Fundus examination reveals severe vitritis and multiple white-yellowish plaques with hemorrhages and exudation in the posterior pole of the right eye. (B) Outer retinal edema is observed on optical coherence tomography of the right eye. (C) Examination of the anterior segment reveals severe inflammation. (D) B-scan ultrasonography confirms vitreous opacification.

On ophthalmologic examination, the visual acuity in the right eye was reduced to hand motion. The left eye was normal. The dilated fundus examination revealed vitritis and multiple white-yellowish plaques in the retina with hemorrhages and exudates in the posterior pole of the right eye (Fig. 1A). On optical coherence tomography (OCT), outer retinal edema of the macula of the right eye was observed (Fig. 1B). The anterior segment examination showed severe inflammation (Fig. 1C), and the intraocular pressure was 12 mm Hg. B-scan ultrasonography confirmed the presence of vitreous opacities (Fig. 1D).

Based on the clinical findings, a provisional diagnosis of EE and CMV retinitis was made on the day of presentation (Day 1). An aqueous humor tap was performed, and the sample was sent for microbiological investigations and viral polymerase chain reaction (PCR): CMV, herpes simplex virus, and varicella zoster virus. But Gram staining, fungus culture, and viral PCR were all negative. Blood tests showed an elevated erythrocyte sedimentation rate (ESR) of 17 mm/h and C-reactive protein (CRP) of 1.74 mg/dL, with CMV IgM and IgG reactive at 7.16 and 250.0 AU/mL, respectively.

**Figure 2.**
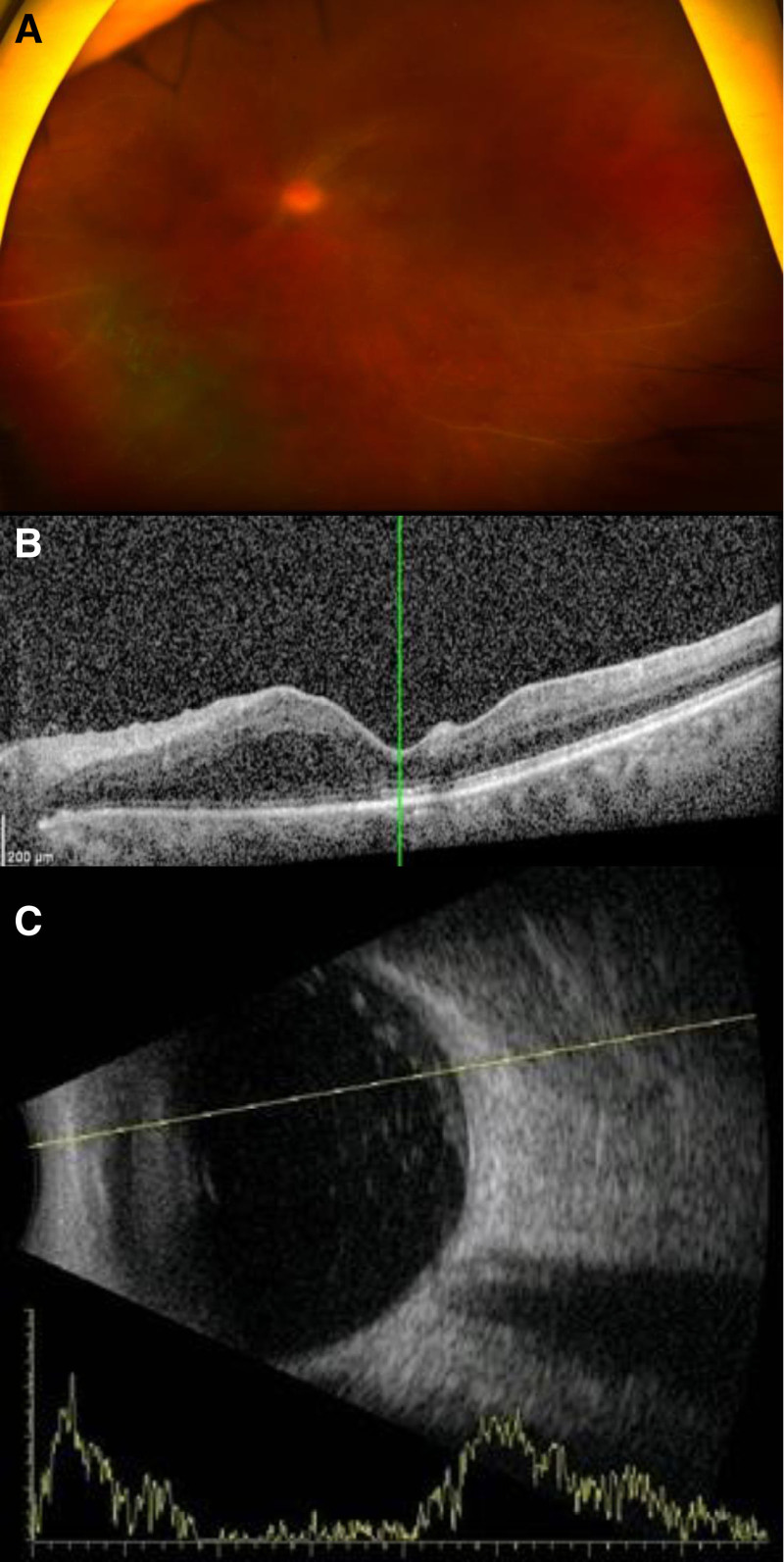
Two days after intravitreal ceftazidime, vancomycin, and ganciclovir injections in the right eye, the patient developed blurred vision in the left eye. (A) Fundus examination reveals vitritis and multiple retinal hemorrhages and exudation in the left eye. (B) Outer retinal edema of the macula of the left eye was observed on optical coherence tomography. (C) B-scan ultrasonography confirms vitreous opacification in the left eye.

Although the aqueous humor tap showed no evidence of infection with bacteria or viruses, the elevated CRP and reactivity to CMV Ig led to empiric antibiotics and antiviral therapy. Intravitreal ceftazidime, vancomycin, and ganciclovir injections were administered to the right eye. However, there was no improvement after 2 days of empiric intravitreal antibiotic and antiviral therapy (Day 3), and the patient developed blurred vision in the left eye, indicating rapid bilateral progression. On examination, the visual acuity in the left eye was reduced to 20/32. The anterior segment examination showed mild inflammation. The dilated fundus examination revealed multiple retinal hemorrhages and exudates in the left eye (Fig. 2A) and mild vitritis (Fig. 2C). On OCT, outer retinal edema of the macula of the left eye was observed (Fig. 2B). Blood tests showed an elevated CRP of 7.06 mg/dL.

**Figure 3.**
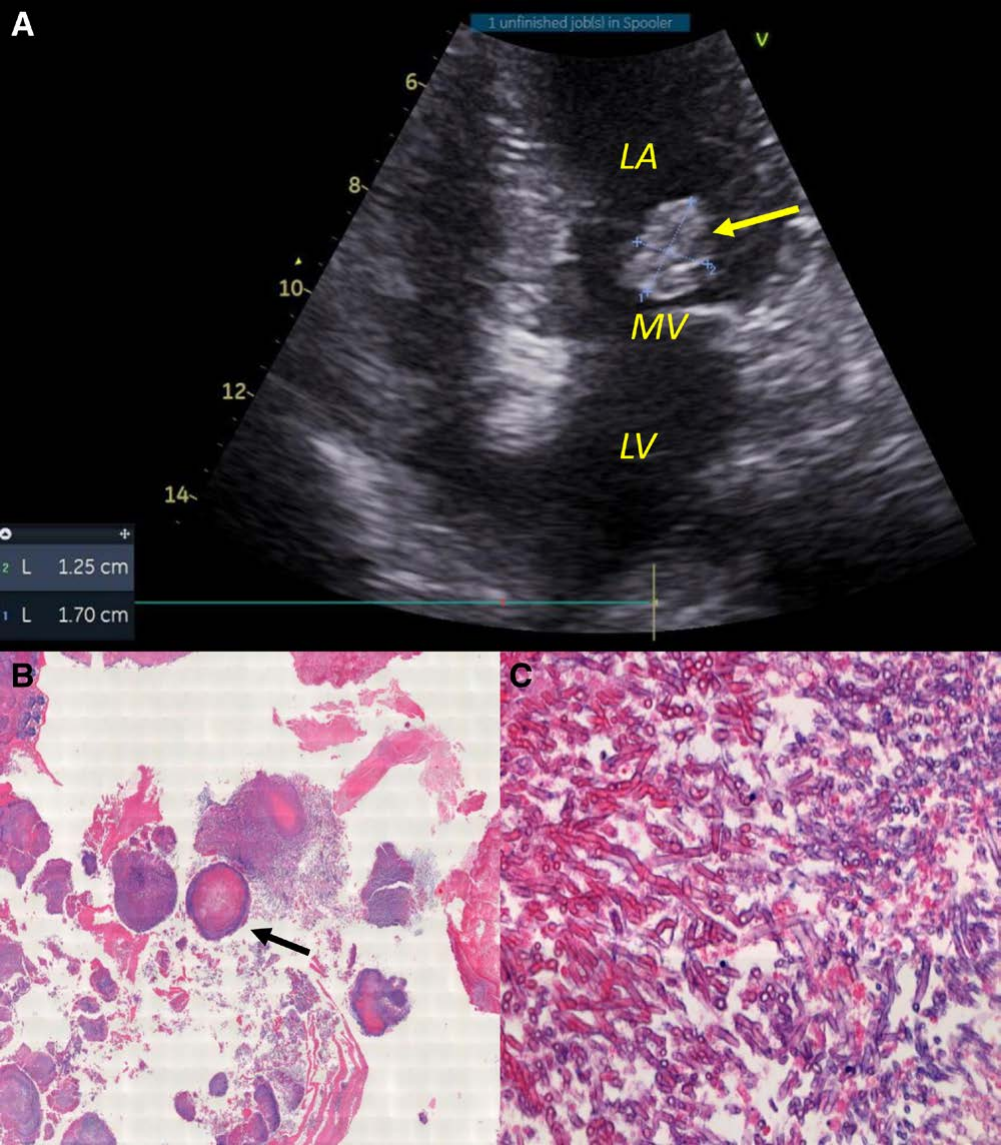
Vegetation observed on transesophageal echocardiography and biopsy results. (A) Transesophageal echocardiography showing a hyper-echoic density mass attached to mitral valve (MV) (yellow arrow). The size of the vegetation was 1.25 × 1.70cm. (B) A fungus ball (black arrow) can be seen (Hematoxylin-Eosin, ×10). (C) PAS (Periodic acid–Schiff) stain highlights thin branching, septate fungal hyphae (×20). LA = left atrium, LV = left ventricle

Due to the rapid bilateral progression, a diagnostic pars plana vitrectomy was performed on Day 3 to both clarify the etiology and reduce vitreous inflammation. A vitreous tap was performed, and the sample was sent for microbiological investigations. Gram staining of the vitreous tap showed the presence of fungal hyphae, and the culture grew *Aspergillus niger (A. niger*). A diagnosis of endogenous fungal endophthalmitis (EFE) was confirmed. Further investigations, including blood cultures – which remained negative for fungal growth – and transesophageal echocardiography, revealed the presence of vegetation attached to the mitral valve (MV) measuring 1.25 cm by 1.70 cm (Fig. 3A). The patient had no fever, chest pain, or dyspnea symptoms, but infectious fungal endocarditis was diagnosed. Intravenous voriconazole (200 mg twice daily) was initiated immediately after the vitreous sample revealed fungal hyphae.

The patient was eventually referred to cardiothoracic surgery for removal of the vegetation, biopsy, and MV replacement. The vegetation was removed, and the MV replacement was successful. Microscopically, a fungal ball (Fig. 3B), morphologically consistent with Aspergillus species, comprising septate, branching hyphae at acute angles (Fig. 3C), were seen. MV replacement and debridement were successful, but she died 2 weeks after surgery due to sepsis from a fungal infection that had spread systemically.

## 3. Discussion

The present case illustrates the difficulties in diagnosis and the possible insidious course of EFE from endocarditis. Aspergillus is an opportunistic infectious fungus known for its high mortality rate. *Aspergillus fumigatus* is reported to be the most common cause of aspergillosis, while *A. niger* is rarely associated with infection. The cases reported were infections in patients who had recently undergone heart surgery or were immunocompromised.^[3]^ Most cases of fungal endocarditis are caused by Candida species, but other fungi, such as Aspergillus, can also cause endocarditis. Aspergillus species account for about 25% of all cases.^[4]^ More than half of cases involving Aspergillus infection are only confirmed at autopsy.^[5]^

EFE is a rare but potentially fatal condition that can be confused with autoimmune uveitis, bacterial, or viral infections. The most common cause is Candida, followed by Aspergillus. In EFE, the incidence of Candida species is reported to be 56%, followed by Aspergillus at 24%.^[6]^ Ocular aspergillosis is more severe than Candida endophthalmitis. Aspergillus favors the subretinal and sub-retinal pigment epithelium space, causing extensive retinitis and choroiditis.^[7]^ In this case, when the patient first presented with decreased vision in the right eye, the findings of retinitis with retinal edema led us to suspect Aspergillus infection, but we could not be sure until the organism was identified.

Delayed diagnosis or initial misdiagnosis of EE is known to be common, with reported rates ranging from 16 to 63%.^[8]^ If EE is strongly suspected, blood and ocular cultures will be performed. However, positive blood cultures ranged from 9.2% to 25.6%, and positive vitreous culture rates ranged from 30% to 70.7%.^[9]^ Because blood and ocular cultures can sometimes be negative, proper recognition of clinical manifestations is important for the treatment of EFE.

Treatment of EFE has been systemic therapy with intravenous antifungals. Commonly used antifungals are amphotericin B, fluconazole, voriconazole, caspofungin, or micafungin. Intravitreal antifungal (voriconazole) injections have also been recommended. Because prolonged waiting for confirmation of a microbiologic culture to initiate antifungal therapy can lead to a worsening of the ocular condition, early vitrectomy plus intravitreal antifungal therapy has recently been reported to yield better visual and structural outcomes than simply vitreous tapping when there is a strong clinical suspicion.^[10]^ In EFE, early vitrectomy removes the fungal elements of the vitreous, which can help treat of the infection and aid in diagnosis.

Although the patient ultimately died due to disseminated fungal sepsis despite appropriate systemic antifungal therapy and surgical intervention, this outcome reflects the aggressive course of *A. niger* infection rather than delayed management. The clinical progression from ocular to systemic dissemination emphasizes the importance of early recognition and initiation of antifungal therapy in suspected EFE, even before definitive microbiologic confirmation.

**Figure 4.**
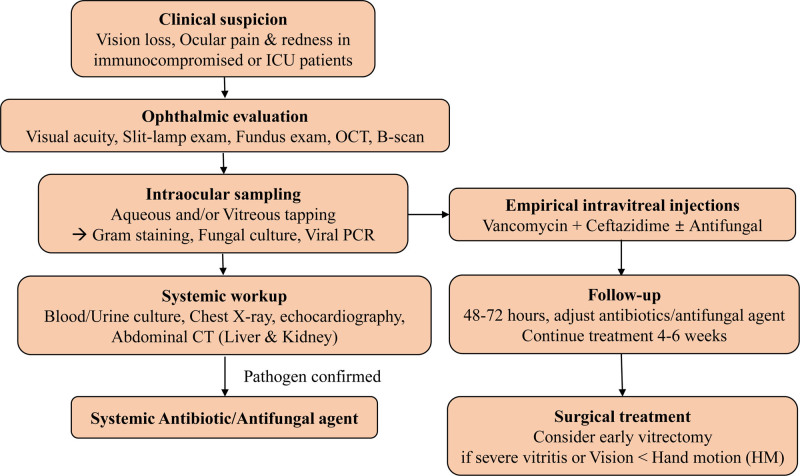
The diagnostic and treatment approach of endogenous endophthalmitis. OCT = optical coherence tomography, PCR = polymerase chain reaction.

While the prognosis for EFE is generally poor, the most important prognosis of this disease is that initial visual acuity has been reported to correlate with final visual acuity.^[11]^ Large-scale studies are needed for conclusive evidence, but conducting such studies for EFE can be challenging. Therefore, based on this case and other references, it’s suggested that in cases of good initial visual acuity, bacteria and fungi should be identified through aqueous humor or vitreous sampling, and empirical intravitreal injections may be considered. And if initial visual acuity is below the ability to count fingers, early vitrectomy to identify the causative agent of infection and reduce the inflammation in the vitreous may be more effective. The diagnostic and treatment approach of EE is shown in Figure 4.

A case of bilateral EE due to endocarditis caused by *A. niger* has not yet been reported. Therefore, this case report suggests that it is necessary to consider EFE in immunosuppressed patients with severe vitritis and rapidly progressive endophthalmitis, even if there are no systemic symptoms except ocular symptoms. It is also important to identify the source of the infection in order to treat it, so if EE is suspected, it is recommended that tests be performed, including aqueous humor and vitreous tapping, blood and urine culture, abdominal imaging, and transesophageal echocardiography.

## Author contributions

**Conceptualization:** Jin-Ho Joo.

**Data curation:** Seowoo Park.

**Formal analysis:** Seowoo Park.

**Funding acquisition:** Jin-Ho Joo.

**Investigation:** Ji Min Kim.

**Methodology:** Ji Min Kim.

**Project administration:** Jin-Ho Joo.

**Supervision:** Jin-Ho Joo.

**Writing – original draft:** Ji Min Kim.

**Writing – review & editing:** Jin-Ho Joo.

## References

[1] Cunningham ET Flynn HW Relhan N Zierhut M. Endogenous endophthalmitis. Ocul Immunol Inflamm. 2018;26:491–5. 29768116 10.1080/09273948.2018.1466561 PMC6448583

[2] Durand ML. Bacterial and fungal endophthalmitis. Clin Microbiol Rev. 2017;30:597–613. 28356323 10.1128/CMR.00113-16 PMC5475221

[3] Kreiss Y Vered Z Keller N Kochva I Sidi Y Gur H. *Aspergillus niger* endocarditis in an immunocompetent patient: an unusual course. Postgrad Med J. 2000;76:105–6. 10644391 10.1136/pmj.76.892.105 PMC1741507

[4] Ellis ME Al-Abdely H Sandridge A Greer W Ventura W. Fungal endocarditis: evidence in the world literature, 1965–1995. Clin Infect Dis. 2001;32:50–62. 11118386 10.1086/317550

[5] Sherman-Weber S Axelrod P Suh B. Infective endocarditis following orthotopic heart transplantation: 10 cases and a review of the literature. Transpl Infect Dis. 2004;6:165–70. 15762934 10.1111/j.1399-3062.2004.00074.x

[6] Smith SR Kroll AJ Lou PL Ryan EA. Endogenous bacterial and fungal endophthalmitis. Int Ophthalmol Clin. 2007;47:173–83. 17450017 10.1097/IIO.0b013e31803778f7

[7] Rao NA Hidayat A. A comparative clinicopathologic study of endogenous mycotic endophthalmitis: variations in clinical and histopathologic changes in candidiasis compared to aspergillosis. Trans Am Ophthalmol Soc. 2000;98:183–93; discussion 193. 11190022 PMC1298225

[8] Maling S King C Davies N. A british ophthalmological surveillance unit study on metastatic endogenous endophthalmitis. Eye (Lond). 2018;32:743–8. 29328066 10.1038/eye.2017.284 PMC5898863

[9] Danielescu C Anton N Stanca HT Munteanu M. Endogenous endophthalmitis: a review of case series published between 2011 and 2020. J Ophthalmol. 2020;2020:8869590. 33149945 10.1155/2020/8869590 PMC7603614

[10] Behera UC Budhwani M Das T. Role of early vitrectomy in the treatment of fungal endophthalmitis. Retina. 2018;38:1385–92. 28541964 10.1097/IAE.0000000000001727

[11] Priluck AZ Huang P Breazzano MP. Outcomes and clinical features predictive of fungal endophthalmitis. Am J Ophthalmol. 2023;251:104–14. 36822571 10.1016/j.ajo.2023.02.011

